# A global media scan on plastic waste using Tobacco Watcher: Opportunities for tobacco control

**DOI:** 10.18332/tid/211359

**Published:** 2025-10-30

**Authors:** Ryan David Kennedy, Emma K. Walker, Harsimrat Kaur, Kevin Welding

**Affiliations:** 1 Institute for Global Tobacco Control, Johns Hopkins Bloomberg School of Public Health, Baltimore, United States

**Keywords:** tobacco, plastics, media scan

**Dear Editor,**

In March 2022, under the auspices of the United Nations Environment Assembly (UNEA-5.2), 175 nations agreed to initiate the development of a UN Treaty to end plastics pollution^1^. At COP10 of the Framework Convention on Tobacco Control (February 2024), the Article 18 Decision recognized that cigarette filters are considered problematic plastics^2^. It is well established that cigarette filters are the most commonly littered item in the world^3^.

We examined international media to assess how plastic waste from tobacco products was being discussed in media stories covering the plastics treaty.

We used Tobacco Watcher (TW), an AI-powered tool that compiles a repository of tobacco-related news stories published in 22 languages from 147 countries^4^. The search included articles in TW inclusive up until 3 January 2025. Within this sample, we used the search terms: ‘plastics treaty’ OR ‘plastic treaty’. We identified the article’s publication date and country of origin. We content-coded each article to identify the type of plastic waste discussed in the article, and the article style (news story or opinion piece). We also summarized how tobacco waste was discussed, and organized these story angles into thematic codes. Articles were coded by the authors, with discrepancies resolved by consensus. Articles published in a language other than English were translated using Google Translate.

The initial search identified 151 articles. After removing duplicates and non-relevant articles, our sample included 32 articles published between May 2022 and May 2024. Articles originated from both high-income countries (HICs, 66%) and low- and middle-income countries (LMICs, 34%). Articles were published in most of the WHO regions of the world, including the Americas (38%), Europe (28%), the Western Pacific (15%), Africa (13%), and Southeast Asia (6%). There were no articles from the Eastern Mediterranean region.

All articles mentioned cigarette filters, and about one-third (31%) also mentioned plastic waste from e-cigarettes (12%), or the plastic packaging waste associated with e-cigarettes/vape products (3%). Most of the articles were classified as news stories (60%). Other articles were opinion/editorial stories (9%) or news stories posted on NGO websites (31%).

Articles contextualized plastic waste from tobacco products as being relevant to environmental health, public health, and/or discussed how there was policy support for addressing tobacco waste. Several articles put forward arguments for banning cigarette filters.

This examination of global media demonstrates that plastic waste from tobacco products is being discussed in articles covering the plastics treaty in most regions of the world; however, the scale of this coverage is relatively small.

In August 2025, discussions in Geneva concluded without an agreed text, and the International Negotiating Committee set up by the UNEA adjourned. However, the issue of plastic pollution remains, and the tobacco control community has ongoing opportunities to ensure national and subnational policies are enacted to address the broad range of environmental and health issues created by plastic wastes from tobacco products.

## Data Availability

Data sharing is not applicable to this article as no new data were created.

## References

[1] Historic day in the campaign to beat plastic pollution: Nations commit to develop a legally binding agreement. The United Nations Environment Programme. March 2, 2022. https://www.unep.org/news-and-stories/press-release/historic-day-campaign-beat-plastic-pollution-nations-commit-develop

[2] TobaccoWatcher. Johns Hopkins Bloomberg School of Public Health’s Institute for Global Tobacco Control. Accessed May 16, 2025. https://tobaccowatcher.globaltobaccocontrol.org/

[3] WHO Framework Convention on Tobacco Control. Decision: (FCTC/COP10(14) Implementation of Article 18 of the WHO FCTC. https://storage.googleapis.com/who-fctc-cop10-source/Decisions/fctc-cop-10-14-en.pdf

[4] UNEP, Secretariat of the WHO FCTC partner to combat microplastics in cigarettes. WHO Framework Convention on Tobacco Control. February 1, 2022. Accessed September 25, 2025. https://fctc.who.int/newsroom/news/item/01-02-2022-unep-secretariat-of-the-who-fctc-partner-to-combat-microplastics-in-cigarettes

